# Anesthetic Routines: The Anesthesiologist's Role in GI Recovery and Postoperative Ileus

**DOI:** 10.4061/2011/976904

**Published:** 2010-12-29

**Authors:** John B. Leslie, Eugene R. Viscusi, Joseph V. Pergolizzi, Sunil J. Panchal

**Affiliations:** ^1^Department of Anesthesiology, Mayo Clinic, 13400 East Shea Boulevard, Scottsdale, AZ 85259-5404, USA; ^2^Department of Anesthesiology, Jefferson Medical College, Thomas Jefferson University, 111 South 11th Street, PA 19107, USA; ^3^Naples Anesthesia and Pain Management Group, Department of Medicine, School of Medicine, Johns Hopkins University, Baltimore, MD 21205-2196, USA; ^4^National Institute of Pain, 4911 Van Dyke Road, Lutz, FL 33558, USA

## Abstract

All patients undergoing bowel resection experience postoperative ileus, a transient cessation of bowel motility that prevents effective transit of intestinal contents or tolerance of oral intake, to varying degrees. An anesthesiologist plays a critical role, not only in the initiation of surgical anesthesia, but also with the selection and transition to effective postoperative analgesia regimens. Attempts to reduce the duration of postoperative ileus have prompted the study of various preoperative, perioperative, and postoperative regimens to facilitate gastrointestinal recovery. These include modifiable variables such as epidural anesthesia and analgesia, opioid-sparing anesthesia and analgesia, fluid restriction, colloid versus crystalloid combinations, prokinetic drugs, and use of the new peripherally acting mu-opioid receptor (PAM-OR) antagonists. Review and appropriate adaptation of these multiple modifiable interventions by anesthesiologists and their surgical colleagues will facilitate implementation of a best-practice management routine for bowel resection procedures that will benefit the patient and the healthcare system.

## 1. Introduction

An anesthesiologist plays a critical role not only in the initiation of surgical anesthesia but also in the selection and transition to an effective maintenance of postoperative analgesia. All patients undergoing bowel resection (BR) experience postoperative ileus (POI), a transient cessation of bowel motility that prevents effective transit of intestinal contents or tolerance of oral intake, to varying degrees [1–3]. Clinically, POI is characterized by delayed passage of flatus and stool, bloating, abdominal distension, abdominal pain, nausea, and vomiting and is associated with an increase in postoperative morbidity and length of hospital stay (LOS) [4]. 

**Figure 1:**
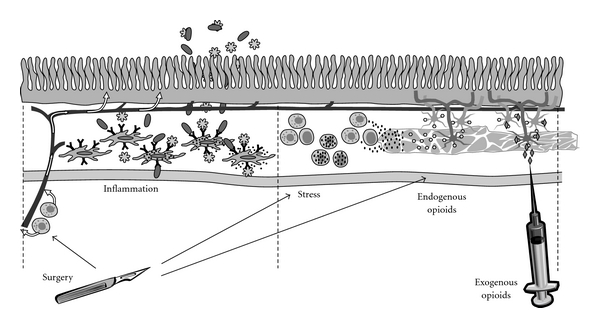
The multifactorial etiology of postoperative ileus (POI). Development of POI is multifactorial. Surgical incision and manipulation of the intestines activate inflammatory and stress responses and endogenous opioids. Mast cells release vasoactive substances diffusing into blood vessels. These substances increase mucosal permeability, allowing entrance of luminal bacteria or LPS into lymphatics or interaction with resident macrophages. Damaged tissue also activates macrophages, increasing expression of proinflammatory genes. Stress causes macrophages to release chemokines and inflammatory cytokines, which attract leukocytes to the intestinal muscularis. Large amounts of nitric oxide and prostaglandins are released, which impair smooth muscle contraction. Endogenous opioids are released, which disrupt GI transit and motility. Exogenous opioid analgesia also disrupts GI motility.

Although the etiology of POI is complex (Figure 1), it is primarily associated with the surgical stress response, an acute inflammatory response associated with manipulation of the bowel, and endogenous opioids secreted within the gastrointestinal (GI) tract in response to surgical trauma [3–7]. Opioid-based analgesia is widely used and considered the standard of care for postoperative pain management [8–12]. Opioids mediate analgesia by binding to mu-opioid receptors in the central nervous system [13]; however, they also bind to peripheral mu-opioid receptors in the GI tract resulting in a disruption of the migrating motor complex and propulsive motor activity associated with GI motility, inhibition of intestinal ion and fluid secretion, and an increase in the overall GI transit time, thereby exacerbating POI [9, 13].

Attempts to reduce the duration of POI have prompted the study of various preoperative, perioperative, and postoperative regimens to facilitate GI recovery. This review focuses on the anesthetic management routines (e.g., opioid-sparing anesthesia and analgesia, epidural anesthesia and analgesia, and use of peripherally acting mu-opioid receptor (PAM-OR) antagonists) that may result in reduced time to gastrointestinal recovery and hospital length of stay. Application of these interventions by anesthesiologists and best practice management routines across the institution may benefit the patient and the healthcare system. 

## 2. Discussion

### 2.1. Can We Modify the Anesthetic Routines to Minimize POI?

The anesthesiologist can contribute to the design and implementation of a best practices routine that defines optimal management routines aimed at accelerating return of GI function and minimization of patient discomfort and costs. One of the first key management decisions the anesthesiologist can help guide is the prevention and management of pain. Because opioid use is clearly linked to adverse GI effects, there is a general consensus that epidural analgesia and other opioid-sparing techniques will improve postoperative GI outcomes. The two most common techniques currently used for management of postoperative pain are epidural analgesia and intravenous patient-controlled analgesia (IV-PCA). Epidural analgesia is generally initiated in the perioperative period and continued throughout the postoperative period for up to 3 postoperative days [8]. Gastrointestinal function was reported in several studies to return 48 to 72 hours earlier in patients receiving thoracic epidural anesthesia and postoperative epidural analgesia compared with patients receiving IV-PCA [10–12]. Thoracic epidurals with local anesthetic (i.e., bupivacaine) significantly reduced duration of POI compared with systemic opioid therapy in patients undergoing abdominal surgery in randomized trials with comparable pain management (*P* < .05) [14–18]. Epidural bupivacaine compared with epidural opioids alone or epidural bupivacaine and morphine combinations significantly reduced the incidence of postoperative nausea and vomiting (*P* < .01) [19], reduced time to first bowel movement [20], and significantly reduced time to GI recovery (*P* < .005) [17] in multiple double-blind studies [18]. Alternatively, when a fast-track postoperative care pathway was used in a recent, randomized controlled trial (*N* = 56) using either thoracic epidural analgesia with bupivacaine and fentanyl for 2 days versus IV-PCA that included opioid-sparing ketorolac, comparable outcomes were obtained for length of stay, pain scores, quality of life, complications, and hospital costs [21].

#### 2.1.1. Opioid-Sparing Analgesia

The strategy to ameliorate negative effects of opioids on the GI tract in the postoperative period (e.g., POI and postoperative nausea and vomiting (PONV)) is often best managed by simply reducing patient exposure to opioids. In a metaanalysis of 52 randomized placebo-controlled trials comparing nonsteroidal anti-inflammatory drugs (NSAIDs), cyclooxygenase-2 (COX-2) inhibitors, and acetaminophen administered in conjunction with morphine after surgery, morphine consumption was reduced 15% to 55% compared with morphine alone. Moreover, NSAIDs administered in conjunction with morphine reduced the incidence of nausea and vomiting from 28.8% to 22.0% compared with morphine alone [22]. In a randomized, double-blind, placebo-controlled study of total hip arthroplasty, addition of COX-2 inhibitors reduced morphine consumption by 40.5% [23]. In 2 prospective, randomized, double-blind studies in patients undergoing colorectal surgery, the addition of ketorolac to morphine IV-PCA had an opioid-sparing effect (patients administered ketorolac received 18.3% to 29% less morphine than patients with comparable pain scores who self-administered morphine alone via PCA) [24, 25]. However, the effect of ketorolac on time to first bowel movement was not always consistent. In 1 study, time to first bowel movement was significantly improved in patients who received ketorolac plus morphine compared with morphine alone, (ketorolac plus morphine, 1.8 days; morphine, 2.4 days;  *P* < .001), and patients who received morphine alone had a 5.25 greater risk of developing POI [25]. However, in the second study the addition of ketorolac did not improve time to first bowel movement (ketorolac and morphine, 1.5 days; morphine, 1.7 days;  *P* < .05) or time to first ambulation (ketorolac and morphine, 2.2 days; morphine, 2.8 days;  *P* < .05) [24]. Additional larger prospective trials are required to determine the benefits of ketorolac on POI. Continuous infusion of lidocaine to augment postoperative analgesia also can reduce the need for opioids, and a metaanalysis review of 8 published trials provides demonstrated benefits in reducing the duration of POI [26].

Gabapentin, a calcium channel modulator, (1,200 mg by mouth 1 hour before surgery) reduced opioid consumption after abdominal surgery compared with placebo in 2 randomized, double-blind trials [27, 28]. Morphine and tramadol consumption after abdominal hysterectomy were reduced in randomized, double-blind, placebo-controlled studies after administration of gabapentin compared with placebo (morphine was reduced by 20 mg;  *P* < .001 ; tramadol was reduced by 149 mg over 24 hours) [27, 28]. Administration of oral gabapentin preoperatively and 24 hours postoperatively reduced morphine consumption by 32% compared with placebo without substantially affecting pain scores in patients undergoing hysterectomy [27]. Fentanyl consumption, as a rescue analgesic, was also reduced in patients undergoing laparoscopic cholecystectomy who received gabapentin in a randomized, double-blind study [29]. However, the effect of this and other opioid-sparing techniques (e.g., off-label use of the alpha-2 agonist dexmedetomidine as a single loading dose or a continuous infusion perioperatively) on POI has yet to be specifically measured. These and other compounds may exert more direct effects on duration of POI, beyond the opioid-sparing benefit, when administered as part of a multimodal analgesic approach. 

Tapentadol, a centrally acting synthetic opioid analgesic, has been approved for use in patients with moderate to severe acute pain [30]. Although the exact mechanism of action is not known, the analgesic effect of tapentadol may be attributed to mu-opioid agonist activity and inhibition of norepinephrine reuptake [30]. The affinity of tapentadol for mu-opioid receptors is 18-times lower than that for morphine; however, tapentadol is only 2 to 3 times less potent in producing analgesia in animal models [30–32]. In a randomized, double-blind, placebo- and active-controlled study, tapentadol 50 mg demonstrated similar pain relief after orthopedic surgery and produced significantly less nausea and/or vomiting compared with oxycodone IR 10 mg [33]. Tapentadol has not been specifically studied for reducing the duration of POI; however, its opioid-sparing analgesic effect could potentially provide benefit in the context of a multimodal pathway.

#### 2.1.2. Epidural Anesthesia

All anesthetics used for induction or maintenance of general anesthesia may depress GI motility [8, 34]. When choosing an anesthetic regimen, the decision to include epidural anesthesia, insertion location of the epidural, selection of inhaled anesthesia agents, use of neuromuscular reversal agents, the extent of the effects of the regimens on the GI tract, and GI recovery should be considered [8, 35]. Differences in these multiple anesthetic variables in previous studies also help to explain the conflicting results of the effects of anesthesia on the GI tract and may affect the duration of POI demonstrated across studies [8, 35].

Location of epidural placement, selection of the epidural infusion mixture, and timing of the first epidural bolus dose are important factors for the risk of developing POI. In abdominal surgeries such as colon resection, the positive effect of an epidural local anesthetic may only be evident when administered in the thoracic region, as it is related to segmental visceral afferent/efferent blockade [8]. In general, thoracic epidural analgesia is associated with improved GI recovery compared with lumbar or systemic analgesia [12]. Numerous studies using lumbar or low-thoracic epidural administration of local anesthetics have failed to demonstrate the positive effects of epidural analgesia on the reduction of the duration of POI [16, 17, 36–38]. Low thoracic (T9 to T12) epidural blockade or dosing of the epidural only after the surgical procedure is completed may not permit sufficient dermatomal blockade of noxious stimuli to eliminate development of a prolonged inhibition of GI motility and facilitate a posttraumatic rapid return to normal function [8].

#### 2.1.3. Perioperative Fluid Administration

Perioperative fluid is administered to meet hourly fluid requirements and to replace fluids lost during surgery [39]. However, excess perioperative fluid administration may lead to edema of the GI tract, resulting in prolonged ileus [40]. Postoperative ileus was reduced in a small, randomized study (*N* = 20) in patients undergoing colonic surgery who were placed under fluid restriction (≤2 L water and 77 mmol sodium per day) during surgery compared with patients who received more liberal fluid management (≥3 L water and 154 mmol sodium per day) [41]. In this study, positive salt and water balance sufficient to cause a 3-kg weight gain delayed return of GI function (median passage of flatus 1 day later and laxation 2.5 days later than patients who received restricted fluids) and hospital discharge (9 postoperative days for patients who received liberal fluids versus 6 postoperative days for patients who received restricted fluids). Furthermore, in a larger randomized study (*N* = 152), patients who received liberal fluids had a significantly longer time to first passage of flatus (Lactated Ringer's 12 mL/kg/hr, 4 days; Lactated Ringer's 4 mL/kg/hr, 3 days;  *P* < .001) or bowel movement (Lactated Ringer's 12 mL/kg/hr, 6 days; Lactated Ringer's 4 mL/kg/hr, 4 days;  *P* < .001) and were discharged from the hospital 1 day later (Lactated Ringer's 12 mL/kg/hr, 9 days; Lactated Ringer's 4 mL/kg/hr, 8 days; *P* = .01) than patients who received restrictive fluids [42]. There were significantly more perioperative complications in patients who received liberal fluids (*n* = 23) compared with patients who received restricted fluids (*n* = 13;  *P* < .05); however, there were more episodes of hypotension in patients who received restricted fluids (36 episodes in 20 patients) compared with patients who received liberal fluids (1 episode in 1 patient) [42].

The use of crystalloid infusions rather than colloids may also predispose patients to excessive intestinal edema [43]. In a small, randomized study (*N* = 18), intestinal edema was demonstrated in patients who received crystalloids during surgery and not in patients who primarily received colloids during surgery [43]. However, while use of fluid restriction rather than liberal fluid administration to replace fluids lost during surgery is recommended, this technique has not entered routine clinical use [39]. Further investigations of fluid restrictions, as well as crystalloids and colloid combinations, are necessary to reach a final consensus on optimal fluid resuscitation and maintenance routines.

#### 2.1.4. Pharmacologic Treatments

Activation of the inducible form of nitric oxide synthase (iNOS) directly modulates intestinal dysmotility after bowel manipulation and is involved in the initiation of intestinal inflammation [5]; therefore, inhibition of iNOS may play a role in reducing duration of POI. In a recent preclinical study, S-methylisothiourea sulfate, an iNOS inhibitor, improved small intestine motility in a canine postoperative model after surgical intestinal manipulation [44]. Further studies are warranted.

Prokinetic agents accelerate gastric emptying or colonic transit and may, therefore, reduce the duration of POI [45]. There are many subclasses of prokinetic agents that theoretically address the multifactorial etiology of POI, including acetylcholinesterase inhibitors, somatostatin analogues, and receptor agonists and antagonists. However, despite promising preclinical data, overall, prokinetic agents have generally failed to demonstrate clinical efficacy in the management of POI, and additional agents are being investigated [46].

Phase I studies have demonstrated that neostigmine, an acetylcholinesterase inhibitor, increases postoperative colonic motility and tone in healthy volunteers and patients undergoing colorectal surgery [47]. Preclinical studies on the somatostatin analogue octreotide demonstrated increased intestinal motility in dogs after small-bowel autotransplantation and amelioration of POI [48, 49]. Receptor-specific compounds (e.g., serotonin and motilin receptor agonists, dopamine and cholecystokinin-1 [CCK-1] receptor antagonists) have also demonstrated some motility-promoting activity [45, 50, 51]. Although multiple serotoninergic agonists (i.e., cisapride, tegaserod, and renzapride) have demonstrated efficacy in promoting GI motility, their beneficial effects are limited because of serious adverse side effects [52–54]. 

Although the antibiotic erythromycin, a motilin receptor agonist, stimulated gastric motility in healthy volunteers [45, 55–57], erythromycin failed to demonstrate any benefit in the management of POI after abdominal surgery [58], and erythromycin did not reduce the rate of nausea, vomiting, or nasogastric tube (NGT) placement in patients after colorectal surgery in a recent randomized double-blind study [59]. Numerous studies have failed to demonstrate any benefit of metoclopramide in the management of POI [60–62]. Despite promising preclinical data, overall, prokinetic agents generally fail to demonstrate clinical efficacy in the management of POI, and additional agents are needed [46].

### 2.2. Are the Opioids the Real Culprits in POI?

All patients undergoing major abdominal surgery are at risk for POI [1–3]. Although the etiology of POI is complex (Figure 1), it is primarily associated with surgical trauma and manipulation of the bowel, inhibitory neural reflexes, secretion of endogenous opioids within the GI tract, and exogenous opioid-based analgesia [4–6, 9, 63–66]. Surgical trauma and manipulation activate inhibitory sympathetic neural reflexes in the GI tract and induce the release of catecholamines, inflammatory mediators, and endogenous opioids. Increased levels of endogenous opioids during surgical trauma to the bowel contribute to impaired GI motility, GI secretions, and POI [5, 13, 67]. 

Opioid-based analgesia is widely used and considered the standard of care for postoperative pain management [9, 13, 68]. Opioids mediate analgesia by binding to mu-opioid receptors in the central nervous system [13]; however, they also bind to peripheral mu-opioid receptors in the GI tract, resulting in a disruption of the migrating motor complex and propulsive motor activity associated with GI motility, inhibition of intestinal ion and fluid secretion, and an increase in the overall GI transit time, thereby exacerbating POI [9, 13]. 

### 2.3. Do the PAM-ORs Really Reduce POI?

Peripherally acting mu-opioid receptor antagonists, such as the FDA-approved alvimopan and investigational methylnaltrexone, do not appear to reverse central opioid action and may give anesthesiologists and surgeons the option of using preemptive opioid analgesics without substantial opioid-associated GI adverse effect [9, 69–79]. Methylnaltrexone has been studied predominantly in patients with opioid-induced bowel dysfunction. More recent clinical studies have reported laxation and reduced oral-cecal transit times after multiple doses of methylnaltrexone in patients with opioid-induced constipation because of methadone maintenance therapy [71, 80]. Methylnaltrexone also reversed opioid-induced constipation in patients undergoing chronic opioid treatment for oncologic pain [81]. Intravenous methylnaltrexone (0.3 mg/kg) accelerated GI recovery (methylnaltrexone, 124 hours; placebo, 151 hours;  *P* = .06) and discharge time (methylnaltrexone, 140 hours; placebo, 165 hours; *P* = .09) in a recent phase II POI trial in patients undergoing segmental colectomy via laparotomy [73]. Opioid consumption was comparable for methylnaltrexone and placebo groups [73]. According to a 2008 Wyeth news press release, a multicenter phase III trial to investigate the efficacy of methylnaltrexone (12 to 24 mg/kg intravenous every 6 hours) in patients undergoing bowel resection via laparotomy did not meet the primary endpoint of decreased time to GI recovery after bowel resection, or its secondary endpoints, including time to discharge eligibility [82]. 

Alvimopan is an oral PAM-OR antagonist with low bioavailability (6%) that was approved by the FDA in 2008 for the management of POI [83]. Alvimopan accelerated time to GI recovery and time to hospital discharge order written compared with placebo in 4 North American phase III trials of POI in patients undergoing bowel resection via laparotomy receiving opioid-based IV-PCA [72, 74, 77–79]. In a fifth phase III trial that was conducted in Europe, the benefits of alvimopan were seen in a post hoc subgroup analysis of patients who received opioid-based IV-PCA for at least the first 48 hours after surgery [76]. Consistent with the role of opioids in the pathoetiology of POI, acceleration of GI recovery for alvimopan versus placebo was not statistically significant in this trial in patients who did not receive opioid-based IV-PCA [76].

Retrospective analyses of the phase III alvimopan trials have demonstrated additional benefits to the patient and the healthcare system. Patients receiving alvimopan 12 mg were less likely to experience POI-related morbidity than patients receiving placebo (odds ratio = 0.44, *P* ≤ .001) [84]. Furthermore, alvimopan patients were significantly less likely to require postoperative NGT insertion and had significantly fewer episodes of prolonged hospital stays or readmission to the hospital for POI symptoms (*P* ≤ .001). These reductions translate into a benefit not only to the patient but to the healthcare system as well. In a recent post hoc economic analysis of the phase III alvimopan trials the mean estimated hospital cost was $879–$977 less for patients who received alvimopan compared with placebo [85].

### 2.4. What Would a Best Practices Plan Look Like? 

Multimodal pathways to accelerate GI recovery and reduce the postoperative length of stay generally include early planned discharge, encouragement of early mobilization, early oral nutrition, and no routine NGT use postoperatively [86, 87]. Patients undergoing these “fast-track” postoperative management protocols consistently have a shorter hospital length of stay than patients receiving traditional care (e.g., NGT in place until bowel sounds return or the passage of flatus) [88–91]. Gum chewing, a type of sham feeding, reduced the time to first flatus and first bowel movement after laparoscopic colectomy in several small, randomized, prospective studies [92–94] and 2 metaanalyses [95]. However, results have not been consistent [96] and a well-controlled, rigorously designed trial is needed to further clarify the effect of gum chewing on GI recovery. Laxatives, as part of a multimodal rehabilitation regimen, were also associated with early GI recovery and hospital discharge in prospective studies [97–99]. However, few randomized controlled studies of laxatives alone (i.e., not as part of a multimodal regimen) have been conducted, and laxatives are generally not used for the management of POI after major abdominal surgery such as bowel resection [50, 100].

Administration of pharmacologic agents initiated in the perioperative period and continued through the postoperative period (e.g., epidural analgesia, PAM-OR antagonists, NSAIDS, and gabapentin) may contribute to faster GI recovery in the postoperative period [17, 29, 101].

### 2.5. How Can We Get Our Institution on Track with the Best Practice Routines for BR and POI Prevention? 

Several key components are important elements to be considered and included in an anesthesia and surgery line of service for BR (Table 1) [11, 27, 35, 43, 50, 83, 86, 89, 95, 102–115]. These suggestions have been shown to contribute to earlier return of GI function either as independent or component variables in similar multimodal management pathways. These factors should be considered for all patients. 

## 3. Conclusions

Postoperative GI dysmotility is the primary determinate of length of hospital stay after abdominal surgery [116, 117]. In the absence of multimodal treatment programs, mean hospital stay after colorectal surgery may be as long as 10 days [118–120]. A study of patients undergoing abdominal surgery revealed that the type and severity of the side effects of pain medications administered were more important to patients than postoperative pain control, highlighting the effect of POI on patient satisfaction [121]. Management of POI and earlier return of GI function may result in improved patient satisfaction and decreased length of hospital stay.

Multimodal techniques reduce the incidence and duration of POI. However, these multimodal techniques require the cooperation of the entire surgical team and collective knowledge of pathways, protocols, and pharmacologic agents with potential to manage POI. It is essential that anesthesiologists play a critical role in the surgical team for providing optimal preoperative, perioperative, and postoperative care. Furthermore, through preoperative discussions and patient assessment, it may be possible to enroll a patient in an individualized multifaceted approach for optimal recovery. It is vital that patients receive high-quality pain relief with minimal impairment of GI function, and that all patients are managed effectively to ensure a rapid return to their normal lifestyle.

##  Conflict of Interests

J. B. Leslie received clinical research funding from Adolor, Wyeth Pharmaceuticals, and Progenics, and honoraria for presentation at CME-sponsored events or meetings funded by grants from GlaxoSmithKline and Adolor or to those organizations. E. R. Viscusi is a consultant for Adolor, and his institution has received grant support from Adolor and Progenics. He is also a speaker for Adolor, GlaxoSmithKline, and Wyeth. J. V. Pergolizzi is a consultant for Adolor, GlaxoSmithKline, and Grünenthal. S. J. Panchal is a speaker for Adolor and GlaxoSmithKline.

## Figures and Tables

**Table 1:** Components of a multimodal management pathway for patients undergoing bowel resection.

Pathway component	Benefit—issues
Preoperative patient education and optimization of medical illness and nutritional status	Reduce preoperative anxiety, minimize perioperative risks, and enhance postoperative recovery [103, 104]
(i) Evaluation and discussion of operative anesthetic plan and perioperative pain management program	Assurance of adequate pain control and selection of appropriate pain management techniques will help with process of controlling sympathetic reflexes, afferent pain and stress-released neuropeptides, and multiple factors contributing to motility inhibition [105]
(ii) Assessment of pain tolerance, history of current and past opiate use and tolerance	Epidural should be thoracic and utilize local anesthetic infusion initiated early during the surgical procedure to minimize any responsiveness [50]
Epidural anesthesia and postoperative analgesia	Insertion and management of epidural must be coordinated with plans for perioperative DVT prophylaxis (e.g., subQ heparin) [11]
IV-PCA	Patients with history of chronic opioid use will likely benefit from use of adjuncts or local anesthetic epidural in combination with IV-PCA to avoid acute withdrawal symptoms [108]
Opioid-sparing adjuncts such as NSAIDs, dexmedetomidine, lidocaine infusion, and gabapentin	Patients with history of opioid intolerance (e.g., PONV, constipation, POI) may benefit from opioid-sparing technique(s) and the addition of PAM-OR antagonists [27, 102, 111, 115]
Patients with planned IV-PCA or opioid tolerance problems evaluated for preoperative initiation of PAM-OR antagonists	PAM-OR antagonists will reverse adverse effects of opioids on GI function without compromising analgesia; PAM-OR antagonists contraindicated in patients on chronic opioids [83]
Preoperative antiemetics and gastric antacids/emptying	Optimize option of early NGT removal at end of procedure; consider 5HT_3_, metoclopramide, and dexamethasone [110, 112]
Preoperative warming blankets and anxiolysis as needed	Reduce intraoperative hypothermia, and reduce preinduction stresses [35]
Laparoscopic surgery	Reduced manipulation and trauma of the bowel leads to less sympathetic activation and inflammation; reduce postoperative pain and associated opioid use [113]
Limited NGT use postoperatively	Utilize intraoperatively but remove at end of procedure as discussed for each case with surgeon; allows resumption of early oral intake [109]
Minimize intraoperative fluids and consider colloid administration	Reduce bowel edema and accelerate GI recovery [43]
Early oral/enteral/sham (gum chewing) feeding initiated POD1	Stimulation of GI hormones [95]
Minimize postoperative opioids	Use of nonopioid analgesics and transition from IV-PCA if used to oral agents when possible with IV opioids used only for breakthrough severe pain [89]
Advancing of diet as tolerated	If clear liquids tolerated on POD1 then advance to soft diet POD2 [86]
Postoperative laxatives	Help to induce bowel movement [114]
Early ambulation	Helps to prevent postoperative complications such as thrombosis, atelectasis, and pneumonia [106]
Discharge planning communication	Will need to work toward multiple components to have patient achieve toleration of adequate oral intake without PONV, adequate pain control, evidence of lower GI activity (stool or gas per surgeon routine), independent ambulation, and adequate support available at home [107]

Abbreviations: DVT: deep vein thrombosis; GI: gastrointestinal; IV-PCA: intravenous patient-controlled analgesia; NGT: nasogastric tube; NSAIDS: nonsteroidal anti-inflammatory drugs; PAM-OR: peripherally acting mu-opioid receptor; POD: postoperative day; POI: postoperative ileus; PONV: postoperative nausea and vomiting; subQ: subcutaneously; TID: three times daily.
